# Translational Investigation of the Therapeutic Potential of Cannabidiol (CBD): Toward a New Age

**DOI:** 10.3389/fimmu.2018.02009

**Published:** 2018-09-21

**Authors:** José A. Crippa, Francisco S. Guimarães, Alline C. Campos, Antonio W. Zuardi

**Affiliations:** ^1^Department of Neurosciences and Behavior, Faculty of Medicine of Ribeirão Preto, University of São Paulo, São Paulo, Brazil; ^2^National Institute for Translational Medicine (INCT-TM; CNPq), São Paulo, Brazil; ^3^Department of Pharmacology, Faculty of Medicine of Ribeirão Preto, University of São Paulo, São Paulo, Brazil

**Keywords:** cannabidiol, CBD, *Cannabis sativa*, anxiolytic, antiepileptic, neuroprotection

## Abstract

**Background:** Among the many cannabinoids in the cannabis plant, cannabidiol (CBD) is a compound that does not produce the typical subjective effects of marijuana.

**Objectives:** The aim of the present review is to describe the main advances in the development of the experimental and clinical use of cannabidiol CBD in neuropsychiatry.

**Methods:** A non-systematic search was performed for studies dealing with therapeutic applications of CBD, especially performed by Brazilian researchers.

**Results:** CBD was shown to have anxiolytic, antipsychotic and neuroprotective properties. In addition, basic and clinical investigations on the effects of CBD have been carried out in the context of many other health conditions, including its potential use in epilepsy, substance abuse and dependence, schizophrenia, social phobia, post-traumatic stress, depression, bipolar disorder, sleep disorders, and Parkinson.

**Discussion:** CBD is an useful and promising molecule that may help patients with a number of clinical conditions. Controlled clinical trials with different neuropsychiatric populations that are currently under investigation should bring important answers in the near future and support the translation of research findings to clinical settings.

## Introduction

The plant *Cannabis sativa* (cannabis) contains more than 100 chemical compounds that share a similar chemical structure, known as cannabinoids. The main psychoactive compound in cannabis is Δ-9-tetrahydrocannabinol (Δ9-THC), responsible for the main effects associated with the use of the plant. Among the many cannabinoids in the plant, our group has focused on CBD, a compound that does not produce the typical subjective effects of marijuana (1).

Since the 1970s, our group has published a number of scientific articles showing the potential therapeutic effects of CBD in different animal models of neuropsychiatric disorders, as well as in clinical trials with humans. We were the first to demonstrate the anxiolytic and antipsychotic effects of CBD in animals, in the 1970s and 1980s, and later in humans, with rather promising results (2). In addition to anxiety and psychosis, basic and clinical research on other therapeutic possibilities of CBD was conducted. Moreover, patentable synthetic analogs of CBD with strong potential for knowledge transfer to the productive sector have recently been developed to offer the possibility of benefits for patients with many health conditions (3).

The aim of the present review is to report the main contributions for the development of the therapeutical potential of CBD in neuropsychiatry, especially performed by Brazilian researchers, which helped to transform the view of CBD from an inactive cannabinoid to a medicine with multiple actions. The studies included here were selected based on searches performed in the online databases PubMed, Web of Science and ScieELO for papers dealing with the therapeutic applications of CBD (“cannabidiol” was used as a keyword).

## Ancient history: an inactive cannabinoid (1940s−1960s)

**Figure 1:**
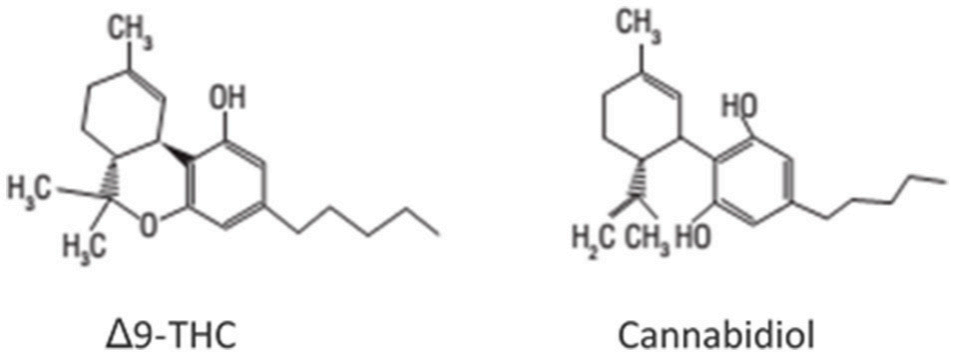
Chemical structures of Δ9-tetrahydrocannabinol (Δ9-THC) and cannabidiol (CBD) (5).

Cannabidiol was isolated from cannabis extracts by Adams et al. (4). However, no further investigation was carried out for almost 25 years, except for a few studies about its isolation. The exact chemical structure of CBD (Figure 1) was elucidated only in 1963 by the group of Professor Raphael Mechoulam, from the Hebrew University of Jerusalem in Israel (6). Throughout the 1960s, the same group was responsible for the determination of the precise structure and stereochemistry of Δ9-THC (Figure 1) and other major cannabinoids. These findings opened a new research field on the pharmacological activity of cannabis constituents. Before the 1970s, only a few pharmacological studies on CBD had been made, which concluded that CBD had no cannabis-like activity, as opposed to Δ9-THC (7).

## The middle ages: an inactive cannabinoid that interacts with Δ9-THC (1970s)

In the early 1970s, several studies reported that CBD was not able to mimic the effects of marijuana, which led to the belief that it would be a non-active cannabinoid. However, this view began to change with the perception that the activity of different cannabis extracts varied widely and that this variation could not be attributed to different levels of Δ9-THC in the samples (8, 9). This finding led to the hypothesis that other cannabinoids in general, and CBD in particular, could interfere with the effects of Δ9-THC.

During this period, a Brazilian research group led by Professor Elisaldo Carlini gave important contributions to the field with their early investigations on the effects of CBD, Δ9-THC, and other cannabinoids. In this context, the investigation of the interaction between the Δ9-THC and CBD began. These studies indicated that CBD had pharmacological effects of its own, which have been investigated since then and led to the current view that CBD actually has a broad spectrum of action (2).

The first study in this line of investigation compared street cannabis samples and a synthetic extract containing the same concentrations of Δ9-THC, CBN, and CBD (10). The authors found that the effects of the samples were not the same in the animals tested, consonant with earlier evidence that the effects of the plant were not only due to its Δ9-THC content (9, 11). Later studies on the interaction between cannabinoids showed that CBD both blocked and potentiated the effects of Δ9-THC in animal tests, depending on the ratio and dose relationship between the two cannabinoids (12, 13). Nowadays, we have evidence that the pharmacokinetic interaction that results in the blockade of P-450 cytochromes by CBD and inhibits the metabolism of Δ9-THC can be overcome by a pharmacodynamic interaction, when the CBD/Δ9-THC ratio is high and/or administration of the two cannabinoids occurs simultaneously or very close in time (14).

Studying the interaction between CBD and Δ9-THC in healthy humans, high oral doses of Δ9-THC provoked anxiety and psychotic symptoms, which were attenuated when CBD was administered together with Δ9-THC (15). These results contributed to support the association of the two cannabinoids in Sativex® (GW-Pharm, UK), a medication used in worldwide for the treatment of pain and spasticity in multiple sclerosis. In addition, these observations contributed to the understanding of the distinct effects of marijuana in different populations, explained by the varying concentrations of the plant's constituents. At the same time, the findings described here suggested that CBD could have anxiolytic and antipsychotic properties and gave rise to a line of research that continues to this day.

## Modern history: CBD effects on anxiety, depression, and psychosis (1980s−1990s)

After a boom in the 1970s, the number of studies on CBD over the two decades that followed became stable, indicating a fall in the interest for the study of the therapeutic actions of this cannabinoid. Although a few groups continued to provide sparse contributions on the subject, much of the production in the field was limited to investigations of the anxiolytic, antidepressant, and antipsychotic properties of CBD performed by Brazilian researchers.

### Anxiolytic action

#### Studies with animal models

As mentioned above, early studies in rodents in the beginning of the 1980s indicated that CBD could interfere with the effects of Δ9-THC and, more specifically, that CBD attenuated the anxiogenic effects of THC on conditioned emotional responses (16, 17). In support to these findings, the same group found that CBD was capable to prevent the anxiogenic and psychosis-like effects induced by elevated doses of Δ9-THC (15). Even though the mechanisms of action of Δ9-THC and CBD were unknown at the time, Zuardi and colleagues had already shown that CBD could potentiate some neuroendocrine effects of Δ9-THC (13). This finding indicated that CBD was not a simple receptor antagonist of Δ9-THC. Together, these results suggested that CBD could be able to cause a physiological antagonism of Δ9-THC effects, having anxiolytic and antipsychotic properties. As shown later, these properties have been confirmed in both laboratory animals and humans.

Initial studies in this line yielded contradictory results. Whereas Zuardi and Karniol et al. (17) found that CBD (10 mg/kg) was able to attenuate conditioned emotional responses in rats, Silveira Filho and Tufik (18) failed to find any effects of CBD (100 mg/kg) in the Geller-Seifter conflict test, considered, at that time, the gold standard among the animal models of anxiety. These apparently contradictory results were explained by Guimarães et al. (19), using a recently introduced test that was sensitive to anxiolytic drugs, the elevated plus maze (EPM). Using this model and covering a full dose-response curve, they showed that in rats CBD does induce anxiolytic-like effects at lower doses (2.5–10 mg/kg) that completely disappear at higher doses (19). As discussed below, this bell-shaped dose-response curve was recently confirmed in humans tested in a clinical model of experimental anxiety.

Since this initial work, several studies have confirmed that CBD decreases anxiety in rodents after either single or repeated administration (Table 1).

**Table 1:** Preclinical studies: anxiolytic-like effects of cannabidiol after systemic administration in rodents.

**Animal model**	**Proposed related disorder**	**Species**	**CBD effects (dose range mg/kg)**	**References**
**SINGLE ADMINISTRATION**				
Elevated plus maze, Vogel conflict test	Generalized anxiety	Rats	Anxiolytic (5–10 mg/kg, bell-shaped dose-response curve)	(19, 20)
Vogel conflict test	Generalized anxiety	Rats	Anxiolytic (10 mg/kg)	(21)
Fear conditioning expression	PTSD and generalized anxiety	Rats	Prevented fear expression (10 mg/kg)	(22)
Fear conditioning extinction	PTSD	Rats	Bidirectional effect (facilitated extinction when the conditioning stimulus was of high-intensity)	(23)
Reconsolidation of aversive memories	PTSD	Rats	Prevented reconsolidation (10 mg/kg)	(24, 25)
Marble burying	OCD	Mice	Anti-compulsive (15–60 mg/kg)	(26, 27)
**REPEATED ADMINISTRATION**				
CUS	Generalized anxiety and depression	Mice	Anti-stress (EPM and NSF; 30 mg/kg daily, 14 days)	(28)
Elevated T-maze	Panic disorder and generalized anxiety	Rats	Panicolytic (5 mg/kg/daily, 21 days)	(29)
Delayed responses to acute stress (predator exposure)	PTSD	Rats	Anxiolytic (5 mg/kg daily, 7 days)	(30)
Marble burying	OCD	Mice	Anti-compulsive (30 mg/kg daily, 7 days)	(26)

*EPM, elevated plus-maze; NSF, novel suppressed feeding; PTSD, post-traumatic stress disorder; OCD, obsessive-compulsive disorder; CUS, chronic unpredictable stress*.

The EPM test is based on innate fear of open and elevated spaces and has been usually associated with general anxiety in humans. Therefore, these investigations were expanded on the effects of CBD to models associated with other anxiety-related clinical disorders, such as panic, post-traumatic stress (PTSD), and obsessive-compulsive disorder (OCD; Table 1). It was also found that CBD has a clear anti-stress effect after either acute or repeated administration, attenuating the behavioral and autonomic consequences of acute restraint stress (31, 32) and the anxiogenic effects of chronic unpredictable stress [CUS—(28)]. These effects could be associated with the antidepressant-like effects of CBD observed in the forced swimming stress, (33, 34), Wistar-Kyoto (35) and bulbectomy models (36).

#### Mechanisms and possible brain sites of CBD's anxiolytic and antidepressant effects

CBD has a complex pharmacology, with several mechanisms proposed to explain its action. Most studies investigating CBD's mechanisms of action have been made *in vitro* (30), but for more than 10 years now, *in vivo* studies using animal models has been investigating how CBD produces its beneficial effects in neuropsychiatric disorders.

**Figure 2:**
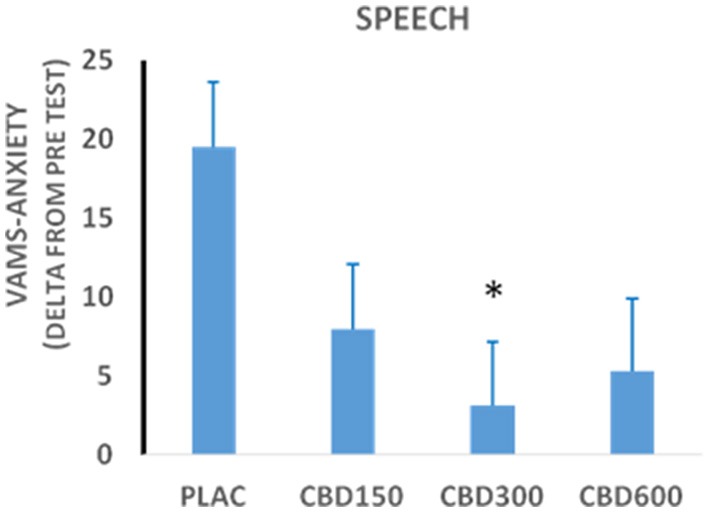
Scores in the anxiety factor of the Visual Analog Mood Scale (VAMS) measured during the performance phase of the Simulated Public Speaking Test in healthy volunteers treated with cannabidiol 150 mg (*n* = 15); 300 mg (*n* = 15); 600 mg (*n* = 12); or placebo (*n* = 15). ^*^Statistically significant differences from the placebo group.

To discover the possible sites underlying CBD effects, a series of studies were performed in rodents using intracerebral drug administration into brain areas related to defensive responses, such as the medial prefrontal cortex (mPFC), dorsal periaqueductal gray (dPAG), bed nucleus of the stria terminallis (BNST), amygdala, and hippocampus. As shown in Figure 2, CBD induced acute anxiolytic effects when injected into the dPAG and BNST (21, 37–41). CBD also modified anxiety-like behaviors in the mPFC, preventing the expression of contextual fear conditioning (41). However, when CBD was tested in the EPM, the picture proved more complicated, as the drug produced opposite effects when injected into the prelimbic or infralimbic regions of the mPFC. In these regions the effects reported varied according not only to the animal model employed but also to previous stress experience (42, 43). Inconsistent results were found following CBD injections into the amygdala after CBD administration (unpublished data). Regarding the hippocampus, although the acute effect of CBD on this structure is still unknown, repeated administration of the drug prevented the anxiogenic effect of chronic stress by facilitating hippocampal neurogenesis (44).

The acute anti-stress effects of CBD also involve the BNST (45) whereas its antidepressant action could be mediated by the hippocampus (44) and mPFC (34).

The pharmacological mechanisms involved in the anxiolytic/antidepressant properties of CBD have also recently been investigated. The acute effects of CBD clearly depend on facilitation of serotonin 5HT1A receptor-mediated neurotransmission in defense-related areas (28, 31, 39, 40, 42, 43, 46, 47). Gomes et al., (48) Not all CBD effects, however, are related to this mechanism (46). Since CBD can decrease the metabolism/uptake of anandamide, a major endocannabinoid, it could also act through this system. Indeed, the acute effects of CBD in the marble burying test and aversive memory reconsolidation were prevented by CB1-receptor antagonists (24, 26). Facilitation of CB1- and CB2-mediated responses, probably due to inhibition of anandamide metabolism, is also involved in the pro-neurogenic effect of CBD (44). Finally, we have also shown that the inverted bell-shaped dose-response curve produced by CBD in several animal models of anxiety/depression is related to the activation of TRPV1 receptors that occurs at higher doses (49). These receptors could increase glutamate release, which would oppose 5HT1A- or CB1-mediated anxiolytic/antidepressant effects.

#### Human studies

According to comprehensive reviews, acute and chronic administration of CBD by various routes (oral, inhaled, intravenous) to healthy volunteers and patients with different clinical conditions did not induce significant adverse effects (50, 51), especially because a conversion of oral cannabidiol to THC seems not to occur in humans (52). These results support previous observations from animal studies according to which CBD appears to be a safe compound for human use over a wide dose range.

The potential anxiolytic effect of CBD was first studied in healthy volunteers using the Simulated Public Speaking Test (SPST). In this model, subjects are asked to speak for a few minutes in front of a video camera while the subjective state of anxiety and its physiological concomitants (heart rate, blood pressure, skin conductance) are recorded. The SPST was shown to be effective in inducing anxiety and sensitive to many anxiogenic and anxiolytic compounds. Through this test, the effects of CBD (300 mg) were compared with those produced by two anxiolytic compounds, ipsapirone (5 mg) and diazepam (10 mg), in a double-blind, placebo-controlled procedure. The findings demonstrated that CBD and the two other anxiolytic compounds all attenuated anxiety induced by the SPST (53).

The apparent validity of the SPST is intrinsic to social anxiety disorder (SAD), since the fear of public speaking and its physiological companions are considered fundamental aspects of this anxiety disorder. Nonetheless, no studies had dealt with the anxiolytic effects of CBD in pathological anxiety until then. We thus investigated this issue in 12 SAD patients treated with CBD 600 mg, 12 SAD patients who received placebo, and 12 healthy subjects who completed the SPST without receiving any medication (54). The group of SAD patients treated with CBD had lower anxiety levels in the performance and anticipatory phases of the test, lower negative self-assessment scores and fewer somatic symptoms compared with SAD patients who received placebo. Furthermore, no significant differences were found between SAD patients treated with CBD and healthy controls, unlike what happened with SAD patients taking placebo.

**Figure 3:**
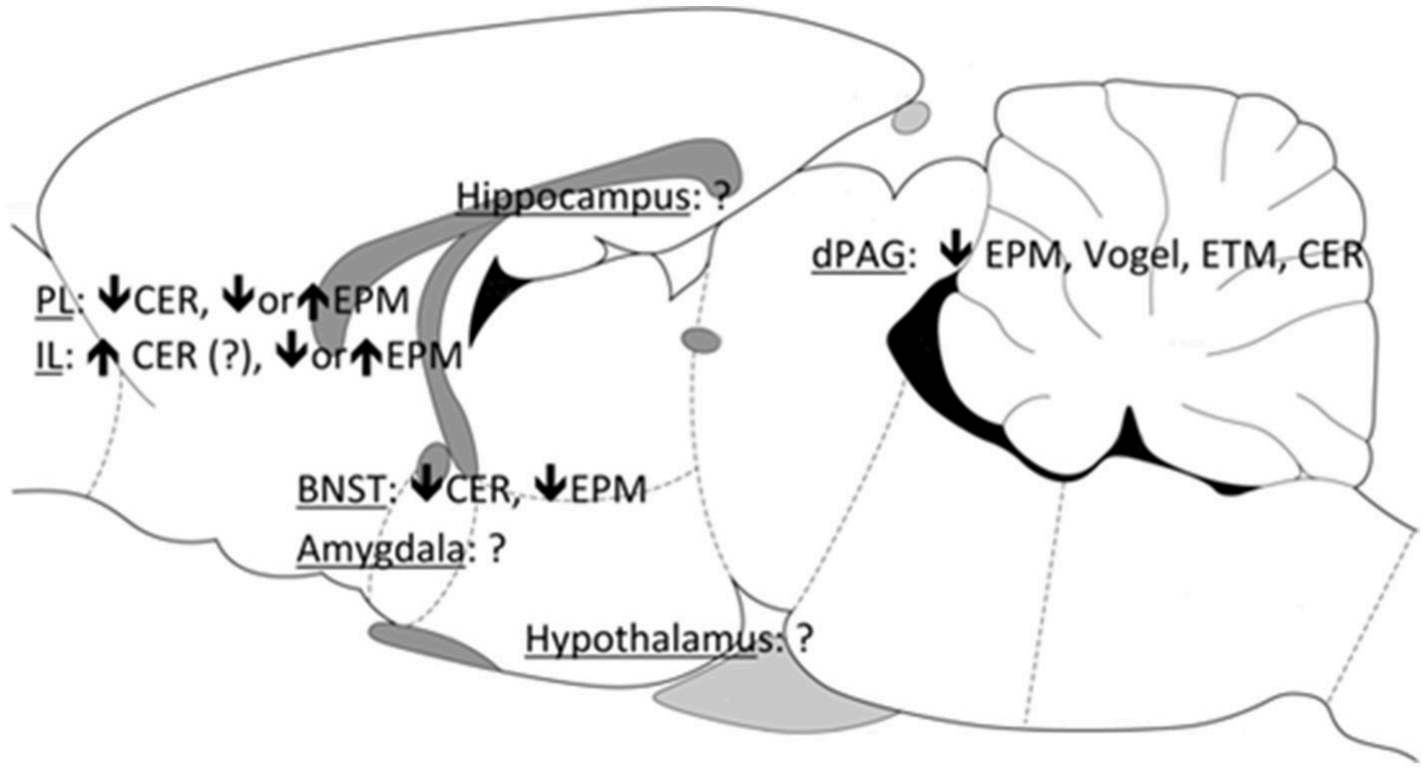
Brain sites associated with the anxiolytic effects of CBD. PL, prelimbic mPFC; IL, infralimbic mPFC; BNST, bed nucleus of the stria terminallis; dPAG, dorsal periaqueductal gray; EPM, elevated plus maze; CER, conditioned emotional response; ETM, elevated T-maze.

We evaluated the effects of different doses of CBD on the SPST in 57 healthy male volunteers divided into four groups (placebo, *n* = 15; CBD 150 mg, *n* = 15; CBD 300 mg, *n* = 15; and CBD 600 mg, *n* = 12) (55). The results confirmed the anxiolytic effect of CBD and the expected inverted “bell-shaped” dose-response curve was observed (Figure 3), in agreement with the findings from animal studies described above. More recently, we further confirmed and expanded this finding with subjects who were assigned to five groups that received CBD (100, 300, and 900 mg), the benzodiazepine clonazepam (1 mg) and placebo and underwent a test of public speaking in a real situation (TPSRS) where each volunteer had to speak in front of a group composed by the other participants. Again, the acute administration of CBD produced anxiolytic effects with a dose-dependent bell-shaped curve in healthy subjects, since the personal anxiety quantities were reduced with CBD 300 mg, but not with the other CBD doses of 100 and 900 mg (56). Therefore, these results highlight the need to establish the accurate therapeutic dose ranges of CBD for each clinical condition.

#### Brain imaging of the anxiolytic effects of CBD

**Figure 4:**
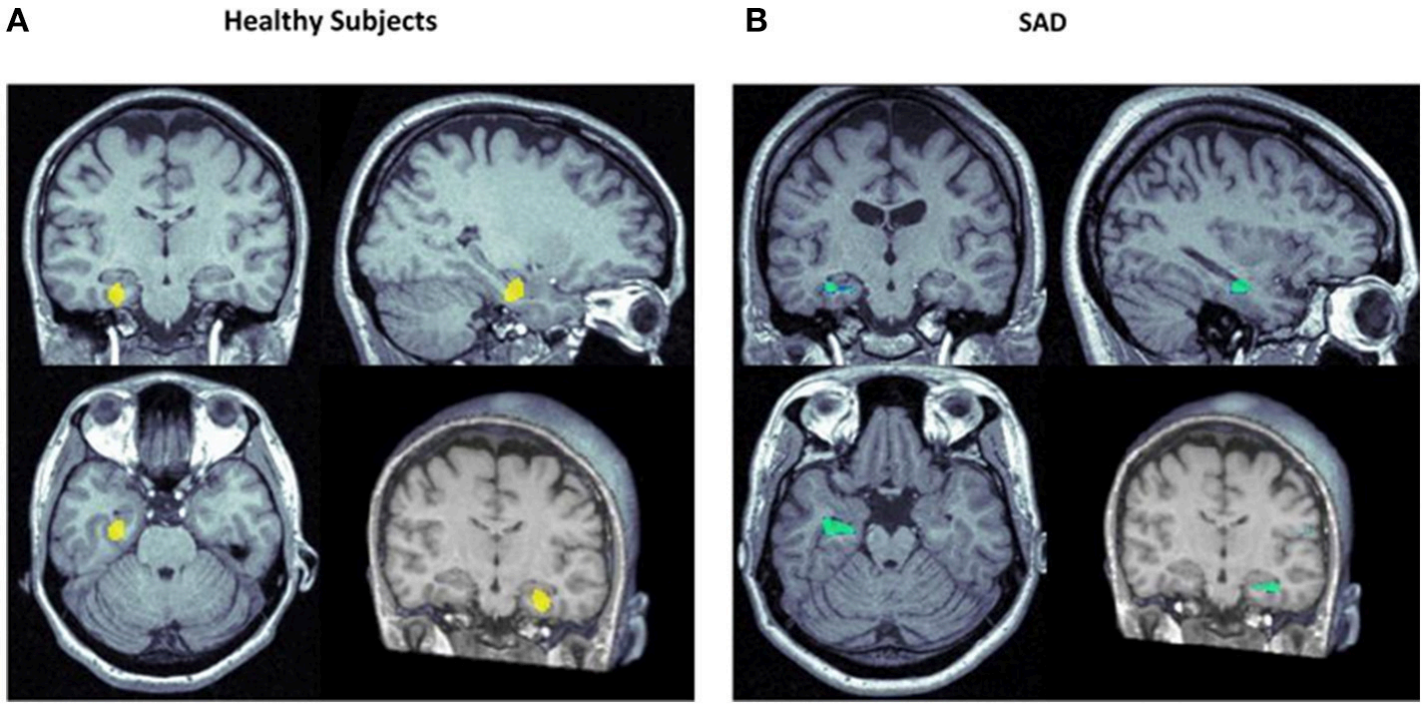
Focus of significantly increased (yellow) and decreased (blue) rCBF in the left hippocampal area in healthy subjects **(A)** (57) and subjects with social anxiety disorder (SAD; **B)** (59) following the administration of CBD vs. placebo.

The first neuroimaging study conducted to investigate the central effects of CBD in humans used single-photon emission computed tomography (SPECT) to evaluate healthy volunteers who received CBD (400 mg) or placebo in two laboratory sessions, 1 week apart, in a crossover, double-blind procedure (57). The whole procedure induced anxiety (58), allowing the investigation of potential anxiolytic effects of CBD. The SPECT results showed an increase in the left parahippocampal gyrus activity and a decrease in left amigdala-hippocampus complex, extending to the left posterior cingulate cortex and the hypothalamus (Figure 4). This brain activity pattern associated with the use of CBD was regarded as compatible with a central anxiolytic effect in these areas.

In a series of neuroimaging studies, functional magnetic resonance imaging (fMRI) was used to investigate the neural correlates of the anxiolytic effects of CBD in 15 healthy subjects (60). This method allowed the acquisition of larger number of images with better spatial and temporalresolution. CBD (600 mg) modulated the patterns of brain activity while subjects processed stimuli depicting intensely fearful faces, attenuating responses in the anterior and posterior cingulate and amygdala. Moreover, this finding had a direct correlation with the concomitant effect of CBD in the modulation of skin conductance responses to fearful stimuli. In a subsequent study, it was also demonstrated that CBD produces its anxiolytic effects by altering prefrontal-subcortical connectivity via amygdala and the anterior cingulate (61).

More recently, we performed the first study to examine the neural correlates of the anxiolytic effects of CBD in a clinical sample (59), using the same protocol, design and dose (400 mg) as in the SPECT study with healthy volunteers described above (57). We found that, when compared to placebo, CBD was able to reduce subjective measures of anxiety without inducing sedation in treatment-naive SAD patients. The anxiolytic effect was associated with reduced activity in the hippocampus, parahippocampal and left temporal gyrus, and increased activity in the posterior cingulated (Figure 4).

Together, these results show that the modulatory effects of CBD in limbic and paralimbic areas are compatible with the effects of anxiolytic compounds on healthy subjects and in patients with anxiety disorders (62–64).

### Antipsychotic-like effects of CBD

#### Studies with animal models

Using classical animal models to evaluate antipsychotic effects, Zuardi et al. (65) made a pioneering investigation and showed that CBD decreased stereotypy induced by dopaminergic agonists, an effect similar to that of haloperidol. As opposed to the latter, however, CBD did not induce catalepsy, a motor impairment associated with antipsychotic extrapyramidal side effects in humans. Moreover, CBD only increased prolactin levels at high doses (greater than 120 mg/kg, Table 2). These effects were similar to those of clozapine, suggesting that CBD could have an atypical antipsychotic profile (65).

**Table 2:** Preclinical studies: antipsychotic-like effects of cannabidiol.

**Model**	**Species**	**CBD effect (dose range in mg/kg)**	**Clozapine effect**	**Haloperidol effect**	**References**
**“DOPAMINERGIC” MODELS**					
Stereotypies induced by DA agonists	Rats	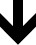 (60)	N.T.	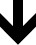	(65)
Prolactin levels	Rats	=( 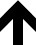 only in high doses: 120–240)	N.T.	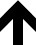	(65)
Catalepsy	Rats, mice	=	=	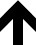	(66)
Hyperlocomotion induced by amphetamine	Mice	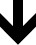 (30–60)	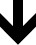	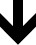	(67)
cFos expression	Rats	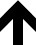 accumbens and mPFC (120)	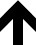 accumbens and mPFC	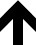 accumbens and striatum	(68)
PPI impairment by amphetamine	Mice	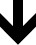 (15–60)	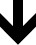	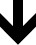	(69)
**“GLUTAMATERGIC” MODELS**					
Hyperlocomotion induced by ketamine	Mice	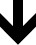 (15–60, bell-shaped dose-response curve)	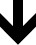	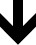	(67)
PPI impairment by MK801 (21 days)^*^	Mice	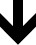 (30–60 daily, for 21 days)	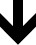	N.T.	(70)

DA, dopamine; mPFC, medial prefrontal cortex; N.T., not tested;

* *repeated CBD also prevented memory (measured with the object recognition test) and social interaction impairment induced by repeated MK801. 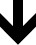
Reduction (or decrease), 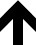
Increase*.

Later, we showed that CBD is also able to decrease hyperlocomotion and pre-pulse inhibition (PPI) impairment induced by amphetamine in both mice and rats (67, 69) (Table 2). The investigations were expanded to glutamatergic-based animal models of schizophrenia, which showed that CBD decreased hyperlocomotion and PPI impairment caused by the glutamate NMDA receptor non-competitive antagonists ketamine and MK801, respectively (67, 71). In the former study, CBD was administered chronically (for 21 days) and was also able to decrease the social impairment and memory deficits induced by the repeated administration of MK801 (70). Again, these effects were similar to those of the antipsychotic clozapine. In fact, mirroring earlier clinical findings (see below), CBD doses that produce antipsychotic-like effects are usually higher than those with anxiolytic action (see Tables 1, 2). Interestingly, in promising a recent study we found that peripubertal treatment with CBD inhibited the development of hyperlocomotion induced by prenatal treatment with poly I:C, indicating that this cannabinoid may have long-lasting properties of a peripubertal treatment as an intervention to prevent psychosis exhibited in the adulthood (72). The translations of these findings to ultra-high-risk subjects to psychosis and in individuals in the early prodromal phases of schizophrenia of great importance, thus studies in this area are underway.

CBD did not induce catalepsy in any of the investigations available to date. In fact, CBD prevented and partially reversed catalepsy caused by haloperidol (48). As discussed below, this result is consonant with the clinical finding that CBD, in addition to blocking L-DOPA-induced psychotic symptoms, may also improve motor impairment in Parkinson's patients (73).

#### Mechanisms and possible brain sites of CBD's antipsychotic effects

In an early work, we observed that CBD produced a pattern of neuronal activation (measured by the expression of the proto-oncogene *cFos*) similar to that of clozapine, but distinct from haloperidol. Whereas the three drugs increased activation in limbic areas, only CBD and clozapine increased activation in the mPFC. Haloperidol, on the other hand, induced a significant increase in cFos expression in the striatum (68). Confirming the involvement of limbic sites in its antipsychotic effects, CBD blocked PPI impairment induced by amphetamine after direct injection into the nucleus accumbens (71).

In respect to the implication of the mPFC in CBD's antipsychotic action, we showed that CBD, as well as the atypical antipsychotic clozapine, prevented the decrease in the expression of parvalbumin (a calcium-binding protein expressed in a subset of GABAergic interneurons) and the increase in FosB/ΔFosB expression in the mPFC after chronic injection of the NMDA antagonist MK-801 (70). Although this latter result seems to contrast with the evidence on cFos mentioned above, FosB/ΔFosB is a marker of neuronal activation after repeated, not acute, stimulation. Finally, both CBD and clozapine prevented the increase in the number of GFAP-positive astrocytes in the mPFC and in the percentage of Iba-1-positive microglia cells with a reactive phenotype in the dorsal hippocampus and mPFCafter repeated administration of MK-801 (71). This result suggests that the anti-inflammatory action of CBD (see (30), for a review), could be related to its antipsychotic properties and is consonant with recent findings that anti-inflammatory drugs such as minocycline could be useful in the treatment of schizophrenia (74).

#### Human studies

In 1995, Zuardi and colleagues published the first case report of a schizophrenia patient treated with CBD (66). A female patient with severe side effects from conventional antipsychotics was treated with CBD up to 1,500 mg/day for 4 weeks and had a significant reduction in the positive and negative Brief Psychiatric Rating Scale (BPRS) symptom scores. In a later trial, three male patients with treatment-resistant schizophrenia were treated with CBD (up to 1,280 mg/day) for 30 days, but only one had a partial improvement (75). The fact that, from the two schizophrenia patients who did not respond, one reduced psychotic symptoms only with clozapine and the other was resistant even to this drug, may justify the findings. None of the patients presented side effects while in treatment with CBD, as previously observed in schizophrenic patients who received CBD and were assessed with electrodermal responsiveness to auditive stimuli and the Stroop Color Word Test (76).

Later, three double blind, controlled clinical trials that investigated the efficacy and tolerability of CBD in schizophrenia patients confirmed our preliminary findings. One study compared the effects of CBD (up to 800 mg/day) to amisulpride in 42 schizophrenia patients treated for 4 weeks (77). Both treatments significantly reduced psychotic symptoms, with no differences between them; however, CBD induced fewer side effects compared to amisulpride.

The antipsychotic effects of CBD were also investigated in first-episode schizophrenia patients treated for 14 days in a crossover, placebo-controlled trial (78). CBD significantly decreased psychotic symptoms after 2 weeks when compared to baseline, although the differences from placebo failed to reach statistical significance.

More recently, in a double-blind trial, patients with schizophrenia were randomized to receive for 6 weeks CBD (1,000 mg/day; *N* = 43) or placebo (*N* = 45) added-on to their existing antipsychotic medications (79). After the treatment, the CBD group presented lower positive psychotic scores and improved cognitive and general illness symptoms. CBD and placebo side-effects were equivalents between groups. Since the antipsychotic effects of CBD do not appear to depend on dopamine receptor antagonism, this compound may indeed represent a new class of treatment for the psychotic disorders in general and schizophrenia, in particular.

The view that CBD could have antipsychotic effects was further supported by our studies in healthy human subjects with artificially induced psychosis (80, 81). In a double blind, placebo-controlled trial, CBD (600 mg) was shown to attenuate depersonalization symptoms induced by the N-methyl-D-aspartate (NMDA) receptor antagonist ketamine, which increases glutamate release at low doses (80). This effect on dissociative symptoms also raised hypotheses of potential therapeutic uses of CBD in conditions such as post-traumatic stress disorder (PTSD), intoxication by cannabis, and some personality disorders.

The management of frequent psychotic symptoms in patients with Parkinson's disease (PD) is regarded as a major challenge for clinicians. It is particularly concerning because (i) the reduction of the doses of antiparkinsonian medications or the addition of conventional antipsychotics worsens motor function; and (ii) atypical antipsychotics may have significant side effects (especially in the neurological and hematological domains) (82). Thus, considering the pertinence of a possible antipsychotic effect of CBD and the lack of effective and safe pharmaceutical management for psychosis in PD, we evaluated the efficacy and safety of this cannabinoid in patients with PD who had psychotic symptoms (73). In an open clinical trial with six PD outpatients, we found a significant reduction in the BPRS and the Parkinson Psychosis Questionnaire (PPQ) scores with CBD (150–600 mg/day) added to the usual treatment. Interestingly, we observed a reduction of both psychotic and motor symptoms during CBD treatment, with no worsening on cognition. These preliminary results suggested that CBD could have potential beneficial effects in PD, which led us to investigate this possibility in greater depth (see below).

#### Brain imaging of the antipsychotic effects of CBD

In a series of collaborative fMRI studies, the effects of CBD were investigated on behavior and regional brain activity in several areas, providing initial clues about its mechanisms and sites of action. Interestingly, opposite brain activation patterns following the administration of CBD (600 mg) and Δ9-THC (10 mg) were observed (62, 83). In contrast to placebo, the psychosis-like effects provoked by Δ9-THC were associated with decreased activation of (i) the dorsal striatum during “oddball” stimuli processing (84); (ii) the ventral striatum and anterior cingulate gyrus during word recall (85); and (iii) the right temporal cortex during auditory processing (86). In all these areas (traditionally associated with psychosis), the effects of CBD on brain activation were contrary to those of Δ9-THC, suggesting that they may be involved in the antipsychotic properties of CBD.

In a subsequent experiment, IV pre-treatment with CBD (5 mg) prevented the psychotic symptoms induced by IV Δ9-THC (1.25 mg) (85). It was thus possible to hypothesize that the opposing effects of these two cannabinoids on brain modulation could be related both to their antagonism but also to the intrinsic antipsychotic effect of CBD. These findings are consistent with the observation that marijuana users of samples containing higher CBD concentrations in addition to Δ9-THC are less likely to experience psychotic symptoms than those who smoke samples without CBD (87).

## Contemporary days: CBD as a compound with a wide spectrum of action (2000s−2010s)

The cloning and description of the CB1 and CB2 cannabinoid receptors in the central system and the subsequent isolation of the endocannabinoids in the early 1990s renewed the interest in the investigation of cannabinoid compounds (2). As a result, there has been a constant increase in the number of investigations on CBD since then, stimulated mainly by discoveries of new therapeutical possibilities of the drug.

### Antiparkinsonian, anti-oxidative, and neuroprotective actions

Although the endocannabinoid system has aroused a promising target in the field of neuroprotection, no trials to date have assessed neuroprotective treatments with CBD for PD. Thus, following an open-trial evaluating the antipsychotic effects of CBD in PD with psychotic features, we tested this cannabinoid in PD patients with no psychiatric comorbidities or dementia (88). We selected 21 PD patients that were assigned to three groups treated with placebo (*n* = 7), CBD 75 mg/day (*n* = 7), and CBD 300 mg/day (*n* = 7). The participants were assessed at baseline and after treatment in regard to motor and general symptoms (UPDRS), quality of life and well-being (PDQ-39), and neuroprotective effects (BDNF levels and H1-MRS). The group treated with CBD 300 mg/day presented significantly lower scores in the PDQ-39. Our findings suggest that CBD may be able to improve general parkinsonism in PD patients with no psychiatric comorbidities. In a series of collaborative animal studies, CBD was unable to prevent or reverse hyperlocomotion induced by the chronic injection of D-AMPH (2 mg/kg). However, we found that CBD seems to have antioxidant and neuroprotective properties, as it increased the levels of brain-derived neurotrophic factor (89, 90). In addition, CBD increased mitochondrial complex and creatine kinase activity (91), reversed oxidative stress parameters (TBARS formation and protein carbonyls) (92), and prevented cognitive impairments (92, 93). In another study, acute and chronic CBD administration (10.0 mg/kg) was able to rescue memory rats treated with iron (94). More recently, we found that CBD reversed iron-induced effects, normalizing hippocampal DNM1L, synaptophysin, and caspase 3 levels in rats and once again suggesting that CBD should be considered as a compound with neuroprotective and memory-rescuing properties (95).

### Neuroprotective and neuroplasticity increasing drug

Our brain continually changes in the course of our lifetime, and the investigation of mechanism involving neuroplasticity offers a great opportunity for the study of maladaptive mechanisms that lead to mental illness and possible new targets for their treatment (96). Neuropsychiatric disorders might be a result of profound changes in mechanism related to brain functions probably involving neuroplasticity (97). For instance, reduced hippocampal volume is observed in patients diagnosed with mood disorders, post-traumatic stress disorder (PTSD), schizophrenia and Alzheimer's Disease (98).

In rodents, exposure to protocol of chronic stressors, that modeling some features of psychiatric disorders symptoms, induce alterations in dendritic remodeling and decrease adult hippocampal neurogenesis (38, 44, 99). Adult hippocampal neurogenesis is complex multi-step process that covers division, survival (not all neurons that divide will survive), migration and differentiation of new cells in the dentate gyrus of the hippocampus (100, 101). In the hippocampus, this process is thought to be determinant in at least some forms of learning and memory. Disturbed adult hippocampal neurogenesis has been recognized as one of the central mechanisms related to the reduction of hippocampal volume reported in patients suffering from mood disorders and schizophrenia. Lower rates of hippocampal neurogenesis were found in post-mortem tissues of schizophrenia patients and depressive patients (102, 103).

Derivatives of *Cannabis sp* have been investigated for their potential effects on neuroplasticity. In 2005, Jiang and co-workers observed that the chronic treatment with a synthetic cannabinoid (HU210) enhanced neurogenesis in rats. Regarding CBD, Wolf et al. (104) were the first to observe that after 6 weeks treatment with a CBD-rich diet, mice exhibited an increased number of neurons positive for the thymidine analog, bromodeoxyuridine (BrdU), in the hippocampus. Results from a different, suggested that besides of promoting adult hippocampal neurogenesis in mice kept basal conditions, CBD (administered intraperitoneally during 15 days) prevented the neurogenic disruption observed in a genetic murine model of Alzheimer's Disease through a peroxisome proliferator-activated receptor γ (PPARγ)-dependent mechanism (105). Results from our group suggested that in chronically stressed mice, CBD prevents stress-induced decreased hippocampal neurogenesis and stress-induced anxiogenesis. However, in transgenic GFAP/Thymidine kinase mice treated with ganciclovir, a model of disrupted adult neurogenesis, CBD was not able to prevent the effects of stress response. These results indicated that the behavioral effect of CBD in stressed mice was partially dependent on the integrity of the neurogenic capacity of the hippocampus.

Recently, Schiavon et al. (106) showed that CBD increased stress-coping behaviors in a behavioral test largely used for the screening of antidepressant drugs, suggested that CBD induces an increased the number of Ki67, BrdU, and double cortin-positive cells in the hippocampus. Interesting, Demirakca et al. (107) suggested that in chronic heavy user of Cannabis, higher THC and lower concentrations of CBD were associated with diminishing gray matter in the hippocampus and reduced cognitive performance, while higher levels of CBD in the consumed Cannabis samples prevented THC induced neurotoxic effects. In their discussion session, authors suggested that a possible mechanism involved in CBD neuroprotective would be its effects in facilitating hippocampal neurogenesis (107).

Studies also suggested that CBD has positive effects on synaptic remodeling. In rats submitted iron overload induced-brain damage, CBD normalized the expression of synaptophysin, an important vesicular protein related to proper synaptic function (95). Moreover, similar to the neurotrophic factor, nerve growth factor, CBD neuritogenesis in PC12 cells, increasing the expression of synaptophysin and synapsin I (108). CBD can modulate intracellular pathways directly connected with synaptic remodeling, such as Erk1/2 and Akt, in distinct types of cancer cell lines (109, 110). Its precise effects in diverse brain regions, however, are still unclear. For example, repeated CBD administration (14 days) decreased phosphorylated forms of Erk1/2 levels in the PFC and improved contextual fear conditioned responses and (111). In chronically stressed mice, chronic CBD administration also promoted dendritic remodeling and increased the expression of Synapsin I/II, PSD95, and p-GSK3β in the hippocampus of rodents submitted to CUS (112).

CBD also has antioxidants activity, acting against the exacerbation of oxygen/nitrogen species (ROS/RNS) production and consequently DNA oxidation, polyunsaturated fatty acids peroxidation and nitration/carbonylation of proteins, leading to cell injury or death (112).

In rat cortical neurons, CBD prevented NMDA-mediated neurotoxicity and oxidative damage in through a cannabinoid receptor-independent mechanism (113). CBD decreases the neuronal damage induced by β-amyloid protein deposit (105); (114–116) and attenuates the depletion of tyrosine hydroxylase, GABA and dopamine levels by modulating the expression of the inducible isoform of NO synthase and reducing the production of ROS-generating NADPH oxidases (114–120). Furthermore, CBD exerts antioxidant activities against toxicity induced different agents, such as amphetamine (89, 116, 121), and attenuates high-glucose-induced mitochondrial ROS production and the expression of pro-inflammatory molecules (122).

In newborn mice, submitted to hypoxic-ischemic brain damage, CBD reduces neuronal damage by reducing the deleterious effects of glutamate, IL-6, TNF alpha, COX-2, and iNOS (123). Using the middle cerebral artery occlusion as method to evaluate ischemia-reperfusion injury, CBD suppressed the reduction of cerebral blood flow after reperfusion, inhibited myeloperoxidase (MPO) activity in neutrophils and reduced the number of MPO immunopositive cells (124). Recently, Mori et al. (125) demonstrated that in mice that underwent to bilateral common carotid artery occlusion, CBD stimulated neurogenesis and, restores dendritic arbor and BDNF levels in the hippocampus. In cultured hippocampal neurons submitted to oxygen-glucose-deprivation/reperfusion, this phytocannabinoid enhances mitochondrial function and reduces oxidative stress (126)

CBD improved cognition, motor activity and BDNF levels in mice administered with thioacetamide, a drug that induces hepatic encephalopathy (127, 128)). In animal models of Parkinson's disease, CBD protects neurons by preventing the tyrosine hydroxylase activity reduction and the dopamine depletion in the substantia nigra (119, 129).

CBD seem to protect neurons from death by enhancing the recycle of old/damaged cell components via facilitations of autophagic action. Autophagy, particularlly macroautophagy, is a lysosomal degradation pathway crucial to recycle injured organelles and promote cell survival, protecting the cell malfunction or death under stress conditions (130). Hosseinzadeh et al. (131), demonstrated that in a model of pilocarpine-induced seizure, the anticonvulsant effects of CBD might involve the activation of hippocampal autophagic machinery (131). Recent findings from our group suggest that chronic CBD treatment increase autophagy in animals submitted to CUS, as observed by its effect in phosphorylated form of mTOR, Beclin-1 and LC3, signaling proteins involved in autophagy induction (112).

### Antiepileptic action

Still in the 1970s, the anticonvulsant effects of CBD were one of the first pharmacological properties of the drug described both in animals (132, 133) and in a preliminary clinical trial in patients led by the same Brazilian group (134). In a recent collaborative animal study, we found that CBD has protective effects not only on seizure control, but also against neuronal death in a model of mesial temporal lobe epilepsy induced by intrahippocampal pilocarpine (135).

We have recently investigated two cases of children with treatment-resistant epilepsy who had full seizure remission, but presented symptoms of intoxication by Δ9-THC and eventual seizure relapse with the use of a cannabidiol-enriched extract (136). When the extract was replaced by pure pharmaceutical-grade CBD, the intoxication signs disappeared and both patients became seizure-free. These observations highlight the importance of GMP/GLP to ensure the development of drugs consistently produced and controlled according to international regulatory standards. More recently, open-blind and double-blind, controlled clinical trials investigated the efficacy, security and tolerability of CBD in children and adolescents with treatment-resistant epilepsy (particularly Dravet and Lennox-Gastaut syndromes) confirmed and expanded these preliminary findings (137–140). Another larger randomized clinical trial in children with treatment-resistant epileptic syndromes using high-quality and reliable CBD is currently underway [NCT02783092, (161)].

### Sleep disorders

One of the most commonly observed effects of CBD at higher doses is sedation (2). REM sleep behavior disorder (RBD) is a parasomnia distinguished by fails in muscle atonia during REM sleep associated with active behavior while dreaming and nightmares. Currently, options for the pharmacological management of RBD are limited. With this in mind, we made an open-trial involving four PD patients with RBD, which is considered a common problem in this movement disorder (141). All patients had a efficient and substantial reduction in the frequency of RBD-related events.

A crossover trial of the acute effects of 300 mg of CBD on the sleep architecture of healthy volunteers has shown that this compound did not interfere with the sleep cycle (55). It is particularly important, since different from anxiolytic and antidepressant drugs such as benzodiazepines and SSRIs, acute administration of an anxiolytic dose of CBD is a safe and appear to preserve the sleep architecture. Thus, CBD may be potentially useful therapeutic option for a wide range of disorders.

### Addiction

There is no approved pharmacological therapy for the treatment of disorders related to the use of cannabis today (142). Based on findings from animal studies, we treated an inpatient with heavy cannabis dependence and episodes of cannabis withdrawal syndrome with CBD, which yielded positive results (143). This finding has been highlighted in Nature Medicine (v. 20, n. 2, pg. 107) as a case of potential success. Likewise, the lack of effective medicines to treat crack cocaine dependence is a clear indication of the need for further research in this field. In a collaborative animal study, we found that CBD protects against cocaine-induced seizures, possibly through activation of the mTOR pathway, with the concomitant reduction in glutamate release (144).

### Mood stabilization

Given the anticonvulsive, anti-anxiety, antidepressant, and antipsychotic actions of CBD described above, we hypothesized that CBD could have a pharmacological profile comparable to that of mood stabilizers. In a collaborative animal study, we initially tested this idea in an animal model of mania induced by chronic injection of D-amphetamine (D-AMPH) at the dose of 2 mg/kg (89). In this model, however, CBD was not able to prevent or reverse the hyperlocomotion induced by D-AMPH. In parallel, we investigated the direct efficacy of CBD in two bipolar affective disorder (BAD) patients in acute manic episodes (145). The patients had no improvement with CBD, which is in line with the negative finding in the animal model of mania and suggests that the drug is not effective in the treatment of manic episodes in BAD. However, new clinical trials assessing CBD effects in depression and anxiety in BAD patients are still necessary.

### Other actions

Over the past 10 years, there has been an exponential increase in the number of publications dealing with the effects of CBD, fostered by the discovery of additional effects of this cannabinoid (2, 64, 83). To take part in the frontline of research in these new areas, Brazilian groups articulated to create the National Institute of Science and Technology for Translational Medicine (INCT-TM), funded by the Brazilian National Council for Scientific and Technological Development (*CNPq*) (146). As a result of this network, we contributed with further evidence on the actions of CBD as a neuroprotector (147), an anti-inflammatory (148, 149), and a drug able to increase sleep periods (150), among other properties (151–157).

## New age: from clinical trials with CBD and its analogs to molecule-targeted therapy

In order to discuss available evidence on CBD and their usefulness and safety for therapeutic use in neuropsychiatric disorders, public hearings and literature reports in different forums and publications occurred worldwide.

Moreover, Professor Raphael Mechoulam, from the Hebrew University of Jerusalem, responsible for the isolation and synthesis of major cannabinoids (including Δ9-THC in 1964 and CBD in 1963) and for the discovery of the endocannabinoid system (CB1 receptor in 1989 and anandamide in 1992), recently contacted the group to develop synthetic fluorinated analogs of CBD (158, 159). These compounds have a strong potential for knowledge transfer to the productive sector, which could enable the commercialization of CBD-based products and offer the possibility of benefits for patients suffering from many of health conditions.

## The battle to define the future: CBD medical uses and law regulations

The panorama of the regulating laws involving human research and medical use of CBD in Brazil has advanced considering in the last 3 years. In January of 2015, the Brazilian regulatory agency ANVISA decided to reclassify CBD from the schedule I regulation (forbidden substances such as THC) to a controlled substance, same category of antidepressants, for instance. Currently, the products containing CBD available for prescription and sales in Brazil are the ones from GW pharmaceutical (Sativex® and Epidioloex®). However, the access of these products for Brazilian patients remain difficult due the high costs of the treatment.

In some countries, however, the medical use of CBD is a reality. In the United States, Cannabidiol is listed as controlled in Schedule I in the Code of Federal Regulations, described as a “derivative” or “component” of marijuana (21 USC 802- Mead et al., (160)- Epilepsy and Behavior). However, since 2012 the medical use of Cannabis, including CBD, is a reality. In some US states, the law includes the “recreational” use of Cannabis. In Canada, Cannabis (marijuana) and its products remains a Schedule II drug under the Controlled Drug and Substances Act. Its production and distribution for medical purposes are regulated. However, the medical use of CBD is permitted under medical prescription. In Europe, the last version of the Cannabis Legislation showed that the use of Cannabis-based drugs is highly regulated. In none of the European Union countries the smoking of Cannabis for medical proposes has been authorized. So far, Sativex® and Epidioloex® are the cannabis-based drugs containing CBD that can be prescribed for patients [European Monitoring Centre for Drugs and Drug Addiction (2017), Cannabis legislation in Europe: an overview, Publications Office of the European Union, Luxembourg].

## Conclusion

In conclusion, the experimental and clinical use of CBD, a compound that does not produce the typical subjective effects of marijuana induced by Δ9-THC, has clearly shown anxiolytic, antiepileptic, and antipsychotic properties, among other effects (2). Since the 1970s, a number of scientific articles showing the potential therapeutic effects of CBD in different animal models of neuropsychiatric disorders and some clinical trials have been published. Recent investigations on the new effects of CBD and its synthetic analogs and on the comprehension of the mechanisms of action of this compounds as well as a better understanding of the endocannabinoid system have emerged. However, new questions appeared regarding the properties of CBD and its synthetic analogs that are currently under investigation, such as the safety and precise dose ranges for each disorder. Therefore, more controlled clinical trials with different and larger neuropsychiatric populations should bring important answers in the near future and support the translation of research findings to clinical settings.

## Author contributions

All authors have been studying the effects of CBD at least for more than 20 years. AZ and JC wrote about the clinical and human data, whereas FG and AC wrote about the animal and pre-clinical data. JC combined both portions, edited, and added some discussion. FG produced the Figures and Tables.

### Conflict of interest statement

AZ, FG, and JC are co-inventors (Mechoulam R, JC, FG, AZ, JH, Breuer A) of the patent “Fluorinated CBD compounds, compositions and uses thereof. Pub. No.: WO/2014/108899. International Application No.: PCT/IL2014/050023” Def. US no. Reg. 62193296; 29/07/2015; INPI on 19/08/2015 (BR1120150164927). The University of São Paulo has licensed the patent to Phytecs Pharm (USP Resolution No. 15.1.130002.1.1). The University of São Paulo has an agreement with Prati-Donaduzzi (Toledo, Brazil) to “develop a pharmaceutical product containing synthetic cannabidiol and prove its safety and therapeutic efficacy in the treatment of epilepsy, schizophrenia, Parkinson's disease, and anxiety disorders.” JC has received travel support from and is medical advisors of BSPG-Pharm. The remaining author declares that the research was conducted in the absence of any commercial or financial relationships that could be construed as a potential conflict of interest.
